# Systemic relational therapy for eating disorders: analysis of family characteristics by means of the SCORE-15

**DOI:** 10.3389/frcha.2025.1669051

**Published:** 2025-11-17

**Authors:** Amanda Bellocci, Giulia Monnetti, Luigi Schepisi, Emanuele Basili, Gianmarco Manfrida, Daniela Tortorelli

**Affiliations:** 1 Psychotherapy School, Centro Studi e Applicazione Della Psicologia Relazionale (C.S.A.P.R.), Prato, Italy; 2 Psychotherapy School, Istituto Italiano di Psicoterapia Relazionale (I.I.P.R.), Rome, Italy; 3 Psychotherapy School, Istituto di Psicoterapia Relazionale (I.P.R.), Rome, Italy

**Keywords:** eating disorders, family functioning, SCORE-15, systemic-relational approach, adolescence

## Abstract

**Introduction:**

The present study, using the SCORE-15 assessment tool, aimed to explore and compare family functioning and therapeutic progress in a group of families with adolescents diagnosed with eating disorders (EDs), compared to a group of families with adolescents presenting other forms of psychopathology. The objective was to analyze the evolution of family functioning throughout the course of therapy, identifying specific characteristics and differences between the two groups. We hypothesized that the sample of families with children diagnosed with EDs (Group A) would present significantly higher initial SCORE-15 scores than the control group (Group B), indicating greater difficulties in communication, relationships, and problem management. We also anticipated variability in scores among members of the same family, particularly between parents and the symptomatic adolescent. Furthermore, therapists' perceptions of the usefulness of therapy and of the degree of family improvement were also investigated.

**Method:**

A retrospective analysis was conducted on a sample of 11 families (Group A) who sought family therapy for an adolescent child diagnosed with an eating disorder at the CSAPR in Prato between 2014 and 2024. Family functioning characteristics were assessed using scores from the SCORE-15 instrument. Group A was compared with a control sample (Group B), consisting of 10 families who initiated family therapy due to other forms of psychopathology in their children during the same time period. The data were analyzed using SPSS Statistics (Statistical Package for the Social Sciences, version 23.0) software. After conducting a descriptive analysis of the demographic variables and constructs of interest in the sample, a comparative analysis of the scores of the participants in the two groups was performed using a Student's *t*-test. Additionally, an analysis of the change in scores over the course of therapy was performed using a Friedman test.

**Results:**

Both groups showed improvement in family functioning over the course of therapy, as indicated by lower SCORE-15 scores. Initially, adolescents in Group A perceived family functioning as more problematic than their fathers did. However, they showed significant improvements during the early phases of treatment, particularly in the Family Difficulties subscale. In Group B, improvement was more evenly distributed among fathers, mothers, and adolescents, with no significant differences in perception. A comparison of the two groups revealed that Group A had a more critical initial condition, especially according to the self-reports of the adolescents, who scored higher than their peers in Group B across all SCORE-15 dimensions. Satisfaction with the therapeutic process increased in both groups, particularly among the fathers and adolescents. However, the mothers, despite having higher initial expectations, showed a less linear pattern of change. Therapists positively evaluated the usefulness of therapy and the potential for family change in both clinical conditions.

**Conclusions:**

The study confirms that, at the outset of therapy, families with adolescents diagnosed with eating disorders (EDs), perceive family functioning as more problematic, particularly from the adolescents' perspective. As we hypothesized, Group A reported higher and more heterogeneous SCORE-15 scores among family members, especially between fathers and adolescents. Nevertheless, both groups benefited from the therapeutic process, showing significant improvements in the quality of relationships and in the management of family difficulties. These results underscore the relevance of a systemic-relational approach in cases of eating disorders and the importance of actively involving the entire family system, paying particular attention to the adolescent's relational experience. The SCORE-15 is confirmed to be a valid and sensitive tool for monitoring change and guiding clinical intervention in family therapy.

## 1 Introduction

Adolescence is a developmental stage characterized by a transformative “crisis” simultaneously involving children and parents within the family system. This crisis should not be interpreted in pathological terms, but rather as a period for restructuring identity and relationships that serves to define the self (1). Thus, adolescence is configured as a phase of existential and relational transition during which individuals face complex developmental tasks, such as differentiation, separation from parental figures, making emotional investments outside the family unit, and individuation (2). This process primarily occurs within the family, which is the primary context of development and the foundational matrix of affective bonds.

The family is conceived as a dynamic, relational system shaped by its own history. It is capable of adapting and reorganizing in response to both internal and external stimuli. From this perspective, an adolescent's growth requires a redefinition of roles, functions, and identities within the family unit (3). The key process structuring this transition is differentiation, a reciprocal movement through which children gradually become independent while maintaining—and in part thanks to—a meaningful emotional bond with their parents. The goal of this transition is to construct an autonomous life project and develop the ability to form stable and mature emotional relationships. This complex emotional and practical process is referred to as emancipation. Much-desired independence is not understood as absolute self-sufficiency but rather as the ability to make autonomous decisions across multiple domains while managing emotional and relational dependencies. The adolescent begins to engage with the external world by questioning the value systems internalized within the family context and facing the challenge of adapting to a broader social reality that often appears inconsistent or incoherent with the domestic one. This oscillation significantly impacts both the family system and the adolescent's still-developing self, which is fragmented and in flux. Affirmations or criticisms from either domain can deeply influence the consolidation of psychological and behavioral structures (4). Psychologically, it is evident that the challenges encountered during this stage of development are particularly complex and delicate. These challenges are also subject to considerable variability. At the same time, parents must redefine their own roles, by transitioning to a phase of social generativity. In this phase, their primary task is to not only raise their children, but also socialize and integrate them into the broader social fabric. This transition entails the capacity to tolerate separation, confront loss, and invest in new relational and identity-based projects, both individually and as a couple. Ultimately, adolescent development emerges as a “shared challenge”—a relational, bidirectional process that actively involves both parents and children in renegotiating emotional bonds, redefining boundaries, and constructing new identities and relational trajectories (5).

The theoretical framework that views adolescence as a process of identity and relational renegotiation within the family system guides the discussion of developmental psychopathology, paying particular attention to the role of family dynamics in its onset and maintenance. It can be asserted that adolescent psychopathology results from the complex interaction of individual, familial, relational, and environmental factors.

Over the past decades, clinical and scientific literature has increasingly recognized the importance of the family context in the development, maintenance, and transformation of psychopathology during adolescence (6–8). For this reason, careful assessment must consider not only the individual but also the family system and the broader context in which symptoms occur. Many approaches—not only systemic ones—incorporate contextual references when evaluating clinical disorders in adolescents. In particular, the systemic-relational approach has provided a fundamental perspective for understanding symptoms as relational manifestations rather than solely individual phenomena (9). Indeed, a child's symptom often represents a form of nonverbal communication that serves a regulatory function within the family system (3, 7, 10). The circulation of unconscious messages and emotions between parents and adolescents must be considered a crucial element of adolescent psychopathology. This perspective explains the contradiction between the distress experienced around symptoms and the ease with which they diminish when environmental conditions change. The disorders that emerge during adolescence typically present as discontinuous, fragmented, and nonspecific subjective emergencies, with their interpersonal significance usually identified at the contextual level. The most common difficulties seen in adolescents primarily fall within the neurotic spectrum and may manifest with varying levels of complexity. Among the most frequent are acting-out behaviors and socialization difficulties, psychosomatic disorders, nonsuicidal self-injury, internet addiction, social withdrawal, and, in more severe cases, suicide or suicide attempts and psychotic onset. In multiproblem families, it is also common to observe psychopathy, sociopathy, and social disorders.

Eating disorders (EDs) play a central role in adolescence due to their incidence and clinical impact (11), spanning a broad spectrum of severity. In a society that is increasingly focused on appearance, the formation of the self becomes a fertile ground on which deeper distress may be expressed through the body, consciously or unconsciously.

The systemic-relational model and family therapy have played a fundamental role in the study and treatment of eating disorders, which can be broadly understood as expressions of a blockage or difficulty in the process of separation and individuation within the family unit (12). The evolution of family therapy in treating eating disorders has undergone several phases, reflecting significant changes in theoretical conceptions and clinical practices. During the 1960s and 1970s, pioneers such as Salvador Minuchin and Mara Selvini Palazzoli introduced innovative approaches to treatment. Minuchin, through structural family therapy, highlighted the importance of family dynamics and dysfunctional boundaries, particularly in families with adolescents diagnosed with anorexia nervosa. Structural family therapy developed interventions for anorexia nervosa from a perspective originally focused on treating psychosomatic families (13, 14). Concurrently, the Milan School developed a systemic approach that regarded symptoms as part of a “relational game” among family members (15, 16). In these cases, the family tends to organize itself around the symptom, which assumes a homeostatic balancing function. This model focused primarily on family communication and interactions, aiming to interrupt dysfunctional cycles through targeted therapeutic interventions. Clinically, specific family dynamics have been identified in families with a member affected by eating disorders, such as enmeshment, overprotection, rigidity, conflict avoidance, and consequent difficulties in separation-individuation processes (12, 14).

However, most traditional studies in this field have focused on analyzing a single eating disorder—anorexia nervosa—without providing a comprehensive overview or inclusive theoretical hypothesis. Indeed, eating disorders today exhibit far more complex and varied characteristics than they did thirty years ago. Currently, these disorders are considered a spectrum of distinct yet related pathologies that tend to overlap and blur boundaries, likely sharing common underlying factors. Similarly, from a systemic-relational perspective, it is conceivable to conceptualize a common grouping that branches out into diverse specificities.

Selvini Palazzoli partially modified her theoretical approach in her later studies on eating disorders (16). She expanded the systemic perspective of eating disorders by including fathers and the trigenerational system, and by correlating various disorders with personality functioning patterns.

Compared to Minuchin's pioneering work, some foundational aspects of structural theory have been revised, especially given the broadened spectrum of feeding and eating disorders. Notably, the concept of rigidity—a typical characteristic of families with psychopathology—has been reconsidered. In families with a member suffering from anorexia nervosa, rigidity appears to be particularly diminished. Loriedo (6) coined a specific term: “pleasant stereotypy”. In these families, rigidity does not consist so much in the repetition of rigid behavioral patterns, but rather in an oscillation that invariably leads to the repetition of extreme and opposing behaviors, with no middle ground and no possibility of reaching and maintaining pre-established and lasting agreements. “*The rigidity of an anorexic family is not the rigidity of a rock but the ebb and flow of water. The therapist's difficulty lies in the fact that when he pushes, the family moves. In this way, he repeatedly has the illusion of leaving a mark on the family structure only to discover that he has been pushed back by the sea*” (ibidem, 28). Regarding the concept of “conflict avoidance,” it is now understood that it is more accurate to highlight an “avoidance of conflict resolution,” which in families with anorexia nervosa manifests as “conflict avoidance,” whereas in those with binge eating disorder it appears as open and recurrent conflict.

From an initial focus on dysfunctional family dynamics and more directive intervention models, there has been a gradual shift toward approaches that emphasize greater collaboration and active support from parents, such as the Maudsley model (17, 18). This transformation has led to more effective treatments centered on the identified patient and the specific characteristics of each family, significantly improving therapeutic outcomes (19). Therapeutic interventions that consider the entire family system in its specificity and uniqueness can indeed facilitate meaningful change, promoting greater psychological and relational well-being for all members involved (20).

Within this framework, the noteworthy work proposed and developed since 1998 by Ugazio (21) and Ugazio and Fellin (22, 23) is also situated. The concept of semantic polarities was introduced as an interpretative tool to understand family representations associated with different psychopathological disorders, highlighting how shared narratives can influence the maintenance or resolution of symptoms. Among these, the semantic polarity identified as characterizing the stories told in therapies for eating disorders is that of power.

Current intervention methods adopt narrative models that integrate recent research and updated approaches. From this perspective, the eating disorder is understood as an expression of a disrupted or blocked narrative within the relational and familial fabric of the patient. Therapeutic work therefore focuses on reconstructing the self-narrative, fostering the emergence of new, more coherent, vital, and shared narratives, in which the symptom can take on new meaning. In this process, the therapist assumes the role of co-author and facilitator of change (24).

The role of family functioning in eating disorders has been extensively studied and empirically supported. More recently, research has begun to converge on integrated multifactorial models of the family and eating disorders, in which the parent-adolescent relationship plays a crucial role (25). Consequently, in recent years, there has been growing scientific interest in analyzing relational and familial factors associated with the onset and maintenance of eating disorders during adolescence. In particular, researchers have increasingly focused on certain relational aspects, such as family functioning and the characteristics and quality of relationships among family members, underscoring the need to measure these dimensions.

Traditionally, diagnostic assessments of eating disorders have relied heavily on instruments focused on symptom severity. Examples include the Eating Disorder Examination Questionnaire (EDE-Q) (26, 27), the Eating Disorder Inventory-3 (EDI-3) (28, 62), and clinical indicators such as Body Mass Index (BMI). These instruments are used to outline the patient's psychopathological profile and physical impairment. However, given the systemic nature of these disorders, it has become essential to not only identify the presence and severity of the disorder, but also to understand the relational context in which it develops and persists.

This focus has led to a growing use of family functioning assessment tools alongside individual symptom severity measures in recent years. Examples include the Family Assessment Device (FAD) (29–31) and the Family Adaptability and Cohesion Evaluation Scales IV (FACES IV) (32–37), which allow exploration of cohesion, flexibility, and perceived relational dysfunction within the family unit, even if these instruments are not specifically designed to evaluate treatment processes (38). Interestingly, in the following study, a significant and positive correlation was found particularly between the SCORE family strenghts dimension and FACES IV both cohesion and flexibility.

Within this context, the present study employs the SCORE-15 (Systemic Clinical Outcome and Routine Evaluation), a self-report questionnaire designed to measure family functioning (39–44). This instrument allows for the assessment of the quality of family functioning and the trajectory of change within the therapeutic setting, providing a more comprehensive and dynamic view of the interaction between individual symptoms and the relational system.

Furthermore, the present research aims to explore and compare the relational, communicative, and adaptive characteristics of families with children exhibiting eating disorder symptoms vs. families with children presenting other forms of psychopathological distress. As in a previous study (45), the aim is to deepen understanding of specific family patterns linked to different symptoms, offering guidance for systemically oriented, tailored clinical interventions.

## 2 Materials and methods

### 2.1 Data collection

The present study was conducted by means of a retrospective analysis of data collected using the SCORE-15, a self-report instrument regularly administered to families and couples at the CSAPR^1^ (Centre for the Study and Application of Relational Psychology) in Prato as an integral part of routine clinical practice. The SCORE-15 is employed for data collection, monitoring therapeutic progress, and internal archiving, independently of specific research projects. For this study, data gathered between 2014 and 2024 were selected and analyzed. The research hypothesis was formulated retrospectively, after the data had already been collected, allowing for a retrospective analysis. The data were analyzed in aggregated and pseudonymized form, and their use fully complied with the ethical principles of research and privacy protection regulations, in accordance with Regulation (EU) 2016/679 (GDPR), with particular reference to Articles 6 and 9 concerning the processing of personal and sensitive data for research purposes.

### 2.2 Research objective and hypothesis

The objective of this study was to explore and compare the therapeutic progress and perceived family functioning in two groups of families:

- Group A—families with adolescents exhibiting symptoms attributable to an eating disorder (ED), according to DSM-5 diagnostic criteria (46);
- Group B—families with adolescents presenting other psychopathologies.

We hypothesized that, at the start of therapy, the sample of family therapies involving an adolescent with eating disorders (Group A) would show heterogeneous scores among family members, particularly between parents and the symptomatic child. Recent studies have indeed found a systematic discrepancy between adolescent patients' and parents' perceptions of family functioning, with adolescents tending to report higher levels of dysfunction (63). Families seeking treatment for adolescent anorexia nervosa often report difficulties in family functioning, with adolescents perceiving the highest levels of impairment (47). For example, a study by Tafà et al. (48) highlights how adolescent girls with anorexia nervosa perceive their families as highly disengaged, poorly interconnected, and rigid; moreover, cohesion and quality of communication are perceived as low. These perceptions are further supported by Cunha et al. (49), who found that patients with anorexia nervosa describe their families as less cohesive and less capable of reinterpreting stressful events to make them more manageable compared to control participants.

Additionally, we hypothesized that families in Group A would initially report significantly higher scores than families in Group B, indicating greater difficulties in communication, relational dynamics, and coping, consistent with the systemic-relational model and other scientific research. For instance, research by Cerniglia et al. (50) shows that families with adolescents diagnosed with feeding and eating disorders—particularly anorexia and bulimia—exhibit significantly lower levels of functioning, with dysfunctional patterns characterized by high rigidity, low cohesion, and communication difficulties. In a comparative study between families of adolescents with anorexia nervosa and a nonclinical control group, low family cohesion emerged as a key feature of anorexic families (51). Family cohesion has been identified as a crucial protective factor: low cohesion levels are associated with greater eating disorder symptoms and interpersonal difficulties, and high rigidity combined with low flexibility increases the risk of eating disorders (52).

Other studies, such as Wisotsky et al. (53), report that the more dysfunctional the perceived family functioning, the greater the severity of eating disorder pathology in daughters diagnosed with anorexia nervosa. Furthermore, research by Berge et al. (54) found that good family functioning, stronger parental connection, and greater parental knowledge of adolescents' activities and whereabouts (e.g., who they are with, what they are doing, where they are) were significantly associated with lower likelihoods of adopting disordered eating behaviors. Conversely, parental psychological control was linked to higher probabilities of such behaviors.

### 2.3 Participants

The total sample in this study consists of 21 families seeking family therapy for symptomatology related to an adolescent. All therapies were conducted by trainee therapists under direct supervision between 2014 and 2024 at the C.S.A.P.R. in Prato. The therapies had an average duration of nine sessions and were carried out based on the “Shared Realities Model”. This model, based on a narrative approach and, developed by Gianmarco Manfrida (24), has its roots in the book by sociologists Berger and Luckmann (55), and has often been used in the therapies on which our research is based. Each family member completed the SCORE-15 questionnaire at the beginning, midpoint (typically the fourth session), and end of therapy as a tool to evaluate therapeutic progress and outcomes.

Our sample was divided into two groups:

- Group A—Eating Disorders (EDs): 11 heterosexual-parent families with adolescents (10 females and 1 male; mean age 16.4 years, SD = 2.11) clinically diagnosed with an eating disorder. The sample included 10 diagnoses of anorexia nervosa and 1 diagnosis of bulimia nervosa. In five cases, the designated patients attended therapy sessions together with their siblings (3 males and 2 females; mean age 17.6 years, SD = 3.43).
- Group B—Other Symptomatology: 10 heterosexual-parent families with adolescents (5 females and 5 males; mean age 17.5 years, SD = 4.27) diagnosed with psychopathological disorders other than EDs. The reasons for therapy referral were extracted from the Clinical Summary Sheets (56): in 4 cases, issues involved adolescent rebellious behaviors, conflict, relational and communication problems; in 4 cases, anxiety disorders with panic attacks; and 2 cases involved depressive disorders. In half of the cases, the designated patients attended sessions with their siblings (2 males and 3 females; mean age 17.2 years, SD = 4.14).

### 2.4 Inclusion criteria

- Request for family therapy due to symptomatology reported in an adolescent
- Therapy conducted and completed at the Centre between 2014 and 2024
- Clinical diagnosis of eating disorder or other psychopathological condition
- Completion of three full administrations of the SCORE-15 at the beginning, midpoint, and end of therapy
- Administration of the SCORE-15 (therapist version) to the supervising trainer

### 2.5 Instrument

The instrument used to assess family functioning is the SCORE-15 (Systemic Clinical Outcome and Routine Evaluation—15-item version) (40, 42, 57–61, 64), a self-report questionnaire grounded in the systemic-relational approach. It consists of 15 items rated by participants on a 5-point Likert scale, divided into three subscales, each exploring a fundamental area of family or relational functioning:

- “*Strengths and Adaptability*”: measures the positive resources of the family system and the ability to adapt to change. This dimension indicates particularly whether family or couple members trust and support each other and solve problems together.
- “*Overwhelmed by Difficulties*”: assesses the level of stress, conflict, and distress perceived within the family system, stemming from a high emotional burden or chronic difficulties that compromise the system's wellbeing.
- “*Disrupted Communication*”: evaluates the quality of communication among family members, including aspects such as clarity, mutual listening, and understanding. This dimension may highlight communication problems that contribute to misunderstandings, conflicts, and emotional disconnection.

The total SCORE-15 score provides an overall measure of perceived family functioning by integrating the three main dimensions. The total score reflects the general level of distress or wellbeing within the family or relational system. It serves as a summary indicator useful for monitoring changes over time, such as before and after therapy. Higher scores indicate more problematic or dysfunctional functioning; lower scores indicate a healthier and more functional system.

The SCORE-15 also includes five additional questions for family members: two open-ended questions on “Family Description” and “Problem Definition” and three Likert-scale questions assessing “Problem Severity”, “Ability to Manage It” and “Usefulness of Therapy.”

Additionally, there is a therapist questionnaire that explores, through two Likert-scale questions, the perceived usefulness of the therapy for the family and the therapist's perception of improvements observed during treatment.

### 2.6 Data analysis

Data were analyzed using the statistical software SPSS Statistics (Statistical Package for the Social Sciences, version 23.0). After conducting an initial descriptive analysis of the sample's demographic variables and key constructs, we performed comparative analyses of scores between participants, between the two groups, and across different therapy phases. Specifically, Student's *t*-test was used to compare SCORE-15 scores and its subscales both among participants and between groups. The test was considered statistically significant at *p* < 0.05.

Finally, the analysis of change trajectories in scores over the course of therapy was conducted using the Friedman test. This test was particularly suitable due to its ability to detect significant differences over time even in small samples and with scores derived from a limited number of subjects, thus ensuring an accurate evaluation of individual progress. Statistical significance was assessed by comparing the test's *p*-value to a predetermined alpha level, typically 0.05. In cases of overall significance, *post-hoc* tests with appropriate corrections for multiple comparisons were conducted to identify the specific therapy phases during which significant differences in scores occurred.

## 3 Results

Below are the results of the comparisons between the average scores of the SCORE-15 and its subscales, divided by groups and family roles (Tables 1–4). Within the current study, regarding the analysis of the adolescents' subgroup, a unified categorization was adopted that did not differentiate between the designated patient and their siblings. This decision was supported by a thorough preliminary analysis aimed at exploring potential significant differences between patients and non-patients across all dimensions examined in the study. The results of this exploration revealed no statistically significant differences, thereby justifying the treatment of the adolescents' group as a single category for the purposes of subsequent analyses.

**Table 1:** SCORE-15 total means' scores on the sample divided by groups and family role.

	Group A		Group B	
	M	SD	M	SD
Total Score-15_T1				
Mothers	37.64	9.92	31.00	7.24
Fathers	31.09	8.13	32.80	7.61
Adolescents	41.07	8.37	30.07	6.10
Total Score-15_T2				
Mothers	36.09	9.14	30.40	7.15
Fathers	32.27	6.75	27.70	5.70
Adolescents	33.91	10.51	27.47	4.61
Total Score-15_T3				
Mothers	33.10	7.81	26.78	7.89
Fathers	29.11	7.75	28.78	8.47
Adolescents	31.25	6.74	29.42	6.39

M, mean; SD, standard deviation.

**Table 2:** SCORE-15 dimensions' mean scores on the sample divided by groups and family role.

	Group A		Group B	
	M	SD	M	SD
Comunication_T1				
Mothers	11.36	2.58	9.50	1.72
Fathers	10.09	3.27	10.50	1.65
Adolescents	12.00	2.51	10.07	2.63
Comunication_T2				
Mothers	10.64	2.38	9.90	1.85
Fathers	10.00	2.49	10.00	2.83
Adolescents	10.64	3.11	9.33	1.95
Comunication_T3				
Mothers	11.10	2.38	8.56	1.94
Fathers	11.11	3.41	10.00	1.94
Adolescents	9.83	1.99	9.42	1.68
Strenghts_T1				
Mothers	12.82	4.88	10.30	3.50
Fathers	11.09	3.59	11.40	3.20
Adolescents	14.71	3.41	10.60	2.56
Strenghts_T2				
Mothers	13.64	4.08	10.40	3.44
Fathers	12.27	3.90	9.30	2.36
Adolescents	13.00	4.92	9.07	2.76
Strenghts_T3				
Mothers	11.50	3.06	9.33	3.67
Fathers	8.78	1.64	9.67	3.35
Adolescents	11.58	4.01	10.33	3.39
Difficulties_T1				
Mothers	13.45	5.01	11.20	5.25
Fathers	9.91	3.96	10.90	4.18
Adolescents	14.36	4.31	9.40	3.52
Difficulties_T2				
Mothers	11.82	4.90	10.10	3.45
Fathers	10.00	3.55	8.40	3.06
Adolescents	10.27	3.93	9.07	2.52
Difficulties_T3				
Mothers	10.50	3.17	8.89	3.76
Fathers	9.22	4.60	9.11	4.17
Adolescents	9.83	3.22	9.67	3.20

M, mean; SD, standard deviation.

**Table 3:** Family satisfactions' mean scores on the sample divided by groups and family role.

	Group A		Group B	
	M	SD	M	SD
Family Satisfaction toward therapy (one item) T1				
Mothers	8.64	1.50	8.10	1.37
Fathers	6.70	3.30	7.30	2.41
Adolescents	8.10	1.37	7.33	2.19
Family Satisfaction toward therapy (one item) T2				
Mothers	8.30	1.49	7.44	2.70
Fathers	8.78	1.30	8.44	1.33
Adolescents	7.44	2.70	7.67	1.88

M, mean; SD, standard deviation.

**Table 4:** Therapist items' mean scores divided by groups and family role.

	Group A		Group B	
	M	SD	M	SD
Therapist's expectations on the usefulness of therapy (one item)				
	8.75	1.04	8.60	1.27
Therapist's satisfaction towards family improvement (one item)				
	3.50	0.54	3.50	0.53

M, mean; SD, standard deviation.

### 3.1 Comparison of mean scores on the SCORE-15 dimensions in Group A (eating disorders)

Analysis of the total SCORE-15 (Table 1) in families from Group A showed a general improvement in family functioning over the course of therapy, with scores decreasing from the first administration (beginning of therapy) to the last (end of therapy). This improvement was observed across all family members, suggesting a broadly positive effect of the therapeutic intervention. Specifically, at the start of therapy, adolescents reported a significantly higher total score compared to fathers [41.07 vs. 31.09; *t*(23) = −2.996, *p* *<* *.05*], indicating a more negative perception of family functioning relative to fathers. No significant differences emerged between mothers and fathers or between mothers and adolescents in total scores at any of the three administrations. Analysis of change trajectories over time revealed that the most marked improvement in the total SCORE-15 score for adolescents occurred between the first and second administrations [*W* = 6, *p* *<* *.05*, *r* = .49].

Scores for each SCORE-15 subscale (“*Strengths and Adaptability*”, “*Overwhelmed by Difficulties*” and “*Disrupted Communication*”) were then analyzed (Table 2). Scores across all three subscales showed a trend toward improvement for all family members, with values decreasing throughout therapy. Fathers in Group A initially reported significantly better scores than adolescents on the “*Strengths and Adaptability*” [*t*(23) = −2.579, *p* *<* *.05*] and “*Overwhelmed by Difficulties*” [*t*(23) = −2.653, *p* *<* *.05*] subscales. At the end of therapy, mothers in Group A showed significantly higher scores on the “*Strengths and Adaptability*” subscale compared to fathers [*t*(17) = −2.372, *p* *<* *.05*], suggesting that, within the parental dyad, mothers perceived less improvement than fathers regarding the family's cohesion, flexibility, and ability to adapt to change.

Furthermore, results showed that the score on the “*Overwhelmed by Difficulties*” subscale reported by adolescents in Group A significantly decreased mainly between the first and second administrations [*W* = 1.50, *p* *<* *.001*, *r* = .53], i.e., during the early phases of therapy. No significant differences over time were observed on the other subscales for different family members, although a general trend toward improvement from the first to the last administration was confirmed.

Regarding satisfaction with therapy (Table 3), there was a general trend of increased satisfaction, especially among fathers and adolescents. Mothers, despite starting with very positive expectations toward therapy, did not show a linear increase in satisfaction over the course of treatment [T1 = 8.64; T2 = 7.64; T3 = 8.30]. Trajectory analyses of satisfaction scores over time did not reveal statistically significant differences among family members in Group A across the three therapy stages.

Therapists rated the utility of the therapy and the potential for change in these families positively (Table 4).

### 3.2 Comparison of mean scores on the SCORE-15 dimensions in Group B (other psychopathologies)

In Group B, family functioning also showed improvement, with a decrease in total SCORE-15 scores from the beginning to the end of therapy, shared by all family members (Table 1). However, no statistically significant differences emerged between fathers, mothers and adolescents in overall scores across the three administrations. Longitudinal analysis revealed that mothers reported significant improvements throughout all phases of the therapy, while fathers and adolescents showed significant improvement between T1 and T2, maintaining these benefits steadily through T3.

The three SCORE-15 subscales demonstrated a general trend toward improvement, without significant differences among family members (Table 2). However, results indicated that mothers reported more significant improvement in the “*Overwhelmed by Difficulties”* subscale at the end of therapy, whereas fathers appeared to show the most significant improvements in this subscale between the first and second administrations—that is, early in the therapeutic process—maintaining the benefits consistently over time. Scores on the “*Strengths and Adaptability”* and “*Disrupted Communication”* subscales did not show significant differences over time for the various family members in Group B, although a general trend of improvement from the first to the last administration was confirmed.

Similarly, satisfaction with therapy increased mainly among fathers and adolescents in this group (Table 3). Mothers began with high expectations, but these did not translate into a corresponding increase in satisfaction over the course of therapy. Trajectory analyses of satisfaction scores over time did not reveal statistically significant differences among family members across the three therapy phases.

As with Group A, therapists working with Group B families expressed very positive evaluations regarding the usefulness of the therapy and the perceived changes within the family (Table 4).

### 3.3 Results of the comparison between the two Groups

The between-group comparison reveals that families in Group A exhibit more problematic family functioning than those in Group B, with significantly higher SCORE-15 scores at both T1 [*t*(69) = 2.968, *p* *<* *.05*] and T2 [*t*(66) = 3.198, *p* *<* *.05*] (Table 1).

Specifically, adolescents from Group A families report significantly higher Total Scale scores compared to those from Group B [*t*(27) = 4.067, *p* *<* *.001*], indicating a more problematic overall picture of family functioning at the beginning of therapy.

On the “*Strengths and Adaptability*” subscale, adolescents in Group A scored significantly higher than their Group B counterparts at both T1 [*t*(27) = 3.695, *p* *<* *.05*] and T2 [*t*(24) = 2.599, *p* *<* *.05*]. On the “*Overwhelmed Difficulties*” dimension, however, they scored significantly higher only at T1 [*t*(27) = 3.403, *p* *<* *.05*]. At the outset of therapy, then, the “*Strengths and Adaptability*” and “*Overwhelmed Difficulties*” subscales appear to be the two most problematic areas for adolescents from families with eating disorders compared to the control group.

Mothers in Group A reported higher scores on the “*Disrupted Communication*” subscale at the end of therapy than mothers in Group B [*t*(17) = 2.535, *p* *<* *.005*].

No significant differences were found between the two groups for fathers across any of the subscales (Table 2).

## 4 Discussion

The results of the present study partially confirm the initial hypothesis, highlighting specific characteristics in the family functioning perceived by families with an adolescent diagnosed with Eating Disorders (EDs), compared to those with other psychopathologies.

According to other studies, even conducted with different instruments [as, i.e., (48, 50)], families in Group A reported significantly higher SCORE-15 scores at the beginning of the therapeutic process, suggesting a more dysfunctional perception of family dynamics, particularly in terms of adaptability, and the management of relational difficulties.

These findings align with recent research (6) that points to rigidity in family functioning and difficulties in managing conflict among families with a member affected by an eating disorder.

A noteworthy finding concerns the significant discrepancy observed in Group A between the perceptions of children and those of fathers, with the former reporting more problematic family functioning. This result confirms previous studies indicating a divergence between the experiences of adolescents with eating disorders and those of their parents, emphasizing the importance of considering individual perspectives within the family, especially during the assessment phase.

Furthermore, the more pronounced improvement observed between T1 and T2 in the children of Group A may reflect the positive impact of the early stages of therapy in reducing perceived distress—an essential element in the early engagement of systemic-relational interventions in these clinical presentations.

Improvements in family functioning were also observed in Group B over the course of therapy, albeit following a more stable trajectory and without significant differences among family members. This may suggest that, in families presenting with symptoms other than EDs, relational distress is less polarized and more evenly distributed among members, and that the perception of dysfunction and relational suffering is less pronounced from the outset.

Another noteworthy finding concerns satisfaction with therapy: in both groups, mothers demonstrated a less linear trend compared to fathers and children. This may reflect greater emotional involvement and higher expectations at the beginning of the therapeutic process, which are sometimes not fully met as therapy progresses. Nevertheless, the absence of statistically significant differences in satisfaction trend suggests a generally stable therapeutic alliance. It is hypothesized that involving the patient more directly in treatment may reduce the centrality—at least partially—of the mother's role, thereby contributing to a more balanced family dynamic.

The between-group comparison indicates that, under equal treatment conditions, families dealing with EDs begin therapy in a more compromised state but still exhibit improvement trajectories comparable to those of families facing other types of symptomatology. This finding supports the effectiveness of family therapy even in more clinically complex contexts and underscores the importance of early systemic intervention in the treatment of eating disorders.

Overall, the results support the validity of the SCORE-15 as a sensitive instrument with which in family functioning throughout the therapeutic process to monitor changes. They also confirm the utility of a narrative-based family approach for adolescents with severe symptoms such as EDs. However, the differences observed among family members and between groups highlight the need for personalized clinical interventions that take into account the specific characteristics of the symptom and the subjective perceptions of each family member.

### 4.1 Research limits

This study presents several limits related to the retrospective nature of the analysis. First, data were originally collected in a clinical setting rather than for research purposes, which made it impossible to systematically control for potentially influential contextual or individual variables. Undocumented variations in the administration of the instrument over the years cannot be ruled out, potentially introducing inconsistencies within the dataset.

The composition of the comparison groups—selected non-randomly and marked by clinical heterogeneity—represents a potential source of bias that limits the generalizability of the findings to other contexts or populations. The groups under comparison (EDs vs. other psychopathologies) may differ in uncontrolled clinical, motivational, or familial characteristics that could influence the scores. Additionally, the lack of homogeneity within the control group may represent another interpretative limit.

The retrospective nature of the study constrained the analysis to pre-existing data, preventing the inclusion of potentially relevant variables that emerged after the research hypothesis was defined or the exploration of specific aspects that had not been originally recorded.

One of the main limits of this study is the relatively small sample size, restricted to a single clinical setting, which reduces the generalizability of the findings to broader clinical or cultural contexts. Another limit concerns the use of a single self-report instrument (SCORE-15), which—despite its validation and alignment with the systemic approach—may be subject to subjective biases, such as social desirability bias.

Despite these limits, the analysis of data collected through the SCORE-15 provides valuable clinical and theoretical insights into family functioning in the treatment of adolescents with Eating Disorders, offering a useful foundation for future studies employing prospective and controlled designs. The use of the SCORE-15 during the diagnostic phase proves helpful in the early identification of critical indicators of family functioning.

### 4.2 Future directions

To address the limits associated with the retrospective design, future studies should adopt a prospective, longitudinal approach, planning data collection with predefined research objectives and standardized procedures. This would allow for more precise control over contextual variables, administration methods, and assessment timelines.

It would also be beneficial to integrate self-report data with additional sources or instruments in order to reduce the risk of subjective bias and obtain a more comprehensive picture of family functioning. The selection of larger and clinically homogeneous samples, ideally recruited from multiple centres, would enhance the generalizability of the findings, and allow for a deeper exploration of diagnostic group differences.

Finally, the inclusion of additional variables—such as socioeconomic factors, other family context characteristics, or the severity of the disorder—could enrich the analysis and support a more detailed understanding of the processes underlying therapeutic change.

## 5 Conclusions

In conclusion, the present study highlights how the use of the SCORE-15 provides a practical and accessible method for analyzing perceived family dynamics. This instrument, characterized by its ease of administration, proves to be particularly valuable both in clinical practice and in research in order to evaluate family functioning in the presence of specific psychopathologies. It supports a deeper understanding of the context in which symptoms emerge, while also allowing for ongoing and systematic monitoring of change processes during treatment.

The findings confirm the importance of a systemic and personalized approach in treating families with adolescents affected by Feeding and Eating Disorders, revealing distinctive patterns in the perception of family functioning compared to other psychopathological conditions. The application of the SCORE-15 has proven effective in detecting and monitoring family dynamics and their evolution throughout the therapeutic process, underscoring the essential role of the family in the healing process and enabling personalized involvement in treatment.

Although the methodological limits—mainly due to the study's retrospective nature—may impact the generalizability of the results, the data nonetheless offer valuable insights for the design of targeted clinical interventions that take into account the differing perspectives of family members, with particular attention to discrepancies between parents and children.

Future studies, employing prospective designs and larger, more homogeneous samples, will be needed to further investigate these findings and gain a deeper understanding of the complexities of adolescent eating disorders.

Moreover, the comparison between families of adolescents with eating disorders and those with other forms of psychological distress highlights the potential of the SCORE-15 to discriminate specific relational patterns associated with different clinical profiles. Although preliminary, these findings emphasize the importance of integrating traditional tools for assessing symptom severity with instruments capable of capturing the systemic and relational dimensions of distress.

Such an integrated approach can inform the development of more effective clinical interventions aimed at fostering meaningful change not only in the individual patient but within the entire family system, thereby promoting shared psychological and relational well-being.

In this regard, it is essential to encourage and support further research in this area, in order to deepen the understanding of family dynamics involved in eating disorders and to develop increasingly targeted and evidence-based intervention models. Research is indeed a vital tool for advancing clinical practice, improving therapeutic outcomes, and effectively supporting families in their care journey.

Therefore, the integration of validated tools such as the SCORE-15 within a multidimensional framework may serve as a key resource for early intervention and therapeutic success, contributing to the overall well-being of not only the adolescent but the entire family unit.

## Data Availability

The raw data supporting the conclusions of this article will be made available by the authors, without undue reservation.

## Footnotes

1 The CSAPR is a postgraduate training institute with a systemic-relational orientation, officially accredited by the Italian Ministry of University and Research (MIUR).
